# A Capacitance-to-Ground Measuring Method for Medium-Voltage Power Grid of a Ship Based on the Combination of High and Low Frequencies

**DOI:** 10.3390/s25237310

**Published:** 2025-12-01

**Authors:** Shuai Wang, Liang Chen, Zhikang Li, Zhenghe Zhao

**Affiliations:** Naval University of Engineering, Wuhan 430030, China15190351714@163.com (Z.L.); 15871769090@163.com (Z.Z.)

**Keywords:** capacitance measurement, high resistance grounding, signal injection method

## Abstract

With the increase in capacity of large ship electric power systems, medium-voltage electric power systems have gradually become an inevitable choice. Among China’s large ships, the neutral point of the power system is usually grounded by high resistance, and its grounding parameters need to be determined, taking the system’s capacitance to ground as a reference. Under different working conditions, the capacitance to the ground of the system will change, which requires online real-time measurement of the capacitance to the ground to provide a basis. However, the current flowing through the distributed capacitance and the capacitance itself cannot be directly measured by measuring instruments. Currently, the most commonly used method is the signal injection method, which can realize the secondary side measurement. This paper analyzed the traditional signal injection methods and found that all these methods are not suitable for real-time measurement of the capacitance to the ground of the medium-voltage electric power system of a ship. Among the current methods, this paper proposes combining the dual-frequency method and the high-frequency method. Through error analysis, for systems with different capacitances to ground, the frequency selection of the dual-frequency method will affect the measurement accuracy. To ensure the measurement accuracy, it is necessary to adopt the principle of “one low frequency + one high frequency”. Therefore, based on the dual-frequency method and the high-frequency method, the paper proposed an improved dual-frequency method, taking a combination method of high frequency and low frequency for capacitance measurement of medium-voltage power systems with high resistance grounding. Then the paper studied the high- and low-frequency selection scheme by simulation comparison and finally determined the frequency selection scheme of 5000/120 Hz. The paper also carried out simulation and experimental verification and finally proved that under the selected frequency selection scheme, the proposed method can accurately measure the capacitance to ground in a medium-voltage power grid with high resistance grounding.

## 1. Introduction

The neutral grounding modes of power grids mainly include ungrounded neutral, neutral grounded via low resistance, neutral grounded via high resistance, and neutral grounded via arc suppression coil [1,2]. In the traditional low-voltage power systems of ships, the ungrounded neutral mode is usually adopted [3] because of its high power supply continuity [4,5]. When a single-phase ground fault occurs, the power systems can still operate continuously for one or two hours. However, with the increase in the capacity and load scale of large ship power systems, the traditional low-voltage AC systems are confronted with problems such as excessive current, huge cable usage, and over-limit fault current. Consequently, medium-voltage power systems have gradually become an inevitable choice.

In 6–10 kV ship power systems, due to the large distributed capacitance between the power grid and the ground, the capacitive current at the single-phase ground fault point tends to be very large, leading to the generation of an unstable intermittent grounding arc. This arc is difficult to extinguish by itself and may cause intermittent arc grounding overvoltage (with a maximum value close to 3.5 times the peak phase voltage [6]). If the fault cannot be eliminated in time, it will further trigger more serious two-phase short circuits or even three-phase short circuits at weak insulation points, thereby causing more terrible hazards to the entire system [4]. Therefore, it is essential to change the grounding mode of the medium-voltage systems [7]. To ensure that the arc at the grounding point can be reliably extinguished by itself, the neutral points of medium-voltage power systems in large ships are mostly grounded via high resistance or arc suppression coils [8]. Regardless of the grounding mode (via high resistance or arc suppression coil), the setting of their parameters requires the measurement of the system’s capacitance to ground as a reference.

In the large medium-voltage ship power systems, the neutral point is usually grounded through a high resistance. In such systems, after relevant calculations and comparisons, the selection of the grounding resistance value at the generator neutral point is very important. The resistance value selection needs to be appropriate; otherwise, if it is too large, when a single-phase grounding fault occurs in the generator stator winding, the insulation of the generator stator winding will be threatened by the arc flash transient overvoltage; if it is too small, the grounding fault current will not be limited within the safe range, which may cause the stator core of the generator to burn out. According to DNV’s Rules for Classification: Ships, “The neutral point of the system is grounded through a resistor, and the resistance value is equal to or slightly less than one-third of the reactance value between one phase and the ground.”

The capacitance to ground of a ship varies under different operating conditions. If this capacitance changes significantly, it is necessary to adjust the resistance value of the grounding resistor based on the capacitance to achieve a better grounding effect. To design a grounding device that can be adjusted in real time according to the operating state of the power grid and use a resistor with variable parameters, online real-time measurement of the capacitance to ground is necessary to provide a basis for parameter selection. However, the current flowing through the distributed capacitance and the capacitance itself cannot be directly measured by measuring instruments such as ammeters [9]. Traditional capacitance measurement methods include direct methods and indirect methods. Nevertheless, due to various shortcomings of direct and indirect methods, such as poor safety and complex operation, the “signal injection method” has been proposed and widely applied.

Luo Ningzhao, Zhang Xiaofeng, et al. proposed that the setting of grounding parameters in the power systems requires the measurement of the ground capacitance as the basis [2,5,6]. Zhang Hailang provided three methods for measuring the capacitance current to the ground of the grid based on the signal injection methods, namely the three-frequency method, the phasor method (dual-frequency method), and the sweep-frequency method (resonance method) [4]. Zhang Wei proposed a method for measuring the capacitance current in the neutral point ungrounded systems [9]. Wang Yuedi proposed an improved method for injecting signals at the neutral point in the systems under the grounding mode of the arc suppression coil [10]. However, these methods have an influence on the measurement accuracy of the voltage transformer’s leakage reactance when measuring large capacitance. Song Xiaoyan proposed the high-frequency method, which can reduce the influence of the voltage transformer’s leakage reactance [11]. Zheng Yifan further clarified the key technologies of the high-frequency method for measuring the ground capacitance current [12], but the method used is still relatively complex, and there is no method for online real-time measurement of the capacitance to the ground of the medium-voltage high-resistance grounded power systems. Therefore, based on the dual-frequency method and the high-frequency method, the paper proposed an improved dual-frequency method, taking a combination method of high frequency and low frequency for capacitance measurement of medium-voltage power systems with high resistance grounding.

As mentioned, the existing signal injection methods are mainly divided into two categories. One is to inject two or three signals of different frequencies into the substation bus through the delta side of the voltage transformer (VT) and establish equations based on the measured feedback signal voltage and signal current to obtain the capacitance current to ground of the distribution network. The other is to inject a variable-frequency signal into the substation bus through the delta side of the VT and connect an inductor in parallel with the delta side of the VT, then calculate the capacitance current to ground of the distribution network by finding the resonant frequency [11]. In practical applications, these methods are referred to as the three-frequency method, dual-frequency method (phase method), and resonance method (frequency sweeping method) [13].

By reviewing relevant literature, the basic principles, advantages, and disadvantages of several current traditional signal injection methods are summarized and compared as follows (Table 1):

In addition, since all signal injection methods require the addition of a voltage transformer (VT) to realize parameter measurement from the secondary side, the influence of the VT on the measurement results also needs to be considered. According to references [6] and [7], the following conclusions can be made (Table 2):

It can be observed that the common problem of different measurement methods is that the leakage reactance of the voltage transformer affects the measurement accuracy when measuring the large distributed capacitance of the systems. In addition, the three-frequency method is only applicable when the system’s capacitance to ground is small [4,11,14,15], while the resonance method is generally used in systems where the neutral point is grounded via an arc suppression coil. Therefore, the applicability of the dual-frequency method for measuring the capacitance to ground of medium-voltage power systems with neutral grounded via high resistance is considered.

Based on the existing signal injection method, this paper analyzes the differences in the measurement principle of the signal injection method for power systems with neutral point high-resistance grounding and those without grounding. It explores the influence of the injection signal frequency and the leakage reactance of the voltage transformer on the capacitance measurement results in the traditional signal injection method. It proposes an improved dual-frequency signal injection method suitable for medium-voltage power systems with neutral point high-resistance grounding, namely the high–low frequency combination method. Theoretical analysis and simulation verification are conducted, and finally, a scale-down experiment is carried out to verify the measurement accuracy and feasibility of this method.

## 2. Error Analysis of the Dual-Frequency Method in Ungrounded Neutral Systems

### 2.1. Basic Principle of the Dual-Frequency Method

**Figure 1:**
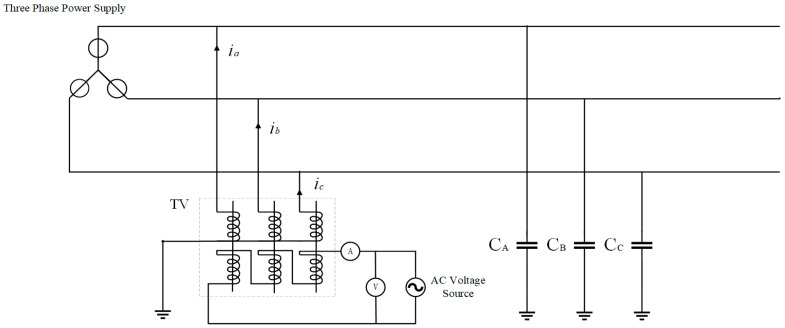
Schematic diagram of ground capacitance measurement in a power grid by the signal injection method.

As shown in Figure 1, the three-phase secondary windings form an open delta terminal. The turns ratio of the VT is *k* (*k* = *n*_1_:*n*_2_, where *n*_1_ and *n*_2_ are the numbers of windings of the primary and secondary windings, respectively). It is assumed that phases A, B, and C are symmetric, and the capacitance to ground of each phase is set as C. A current *i*_0_ with a constant magnitude (effective value) is injected into the open delta side of the VT, and *i*_1_, *i*_2_, and *i*_3_ are the excitation currents of the voltage transformer, while *i_a_*, *i_b_*, and *i_c_* are the currents obtained by converting *i*_0_ to the primary side. Then

(1) $$\left\{\begin{array}{l} n_{1}(i_{a}-i_{1})=n_{2}i_{0}\\ n_{1}(i_{b}-i_{2})=n_{2}i_{0}\\ n_{1}(i_{c}-i_{3})=n_{2}i_{0} \end{array}\right.$$

In the case where the influence of the excitation branch is not considered, *i*_1_ = *i*_2_ = *i*_3_ = 0. Express (1) in phasor form:

(2) $$\left\{\begin{array}{l} n_{1}{\dot{I}}_{a}=n_{2}{\dot{I}}_{0}\\ n_{1}{\dot{I}}_{b}=n_{2}{\dot{I}}_{0}\\ n_{1}{\dot{I}}_{c}=n_{2}{\dot{I}}_{0} \end{array}\right.$$

The induced currents in phases A, B, and C on the primary side are approximately equal, and their magnitudes are determined by the injected current *I*_0_. Thus

(3) $${\dot{I}}_{a}={\dot{I}}_{b}={\dot{I}}_{c}={\dot{I}}_{0}/k$$

**Figure 2:**
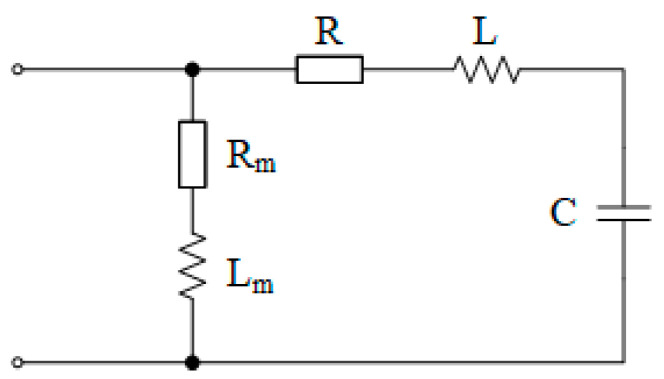
Single-phase equivalent diagram of the primary side in the signal injection method.

**Figure 3:**
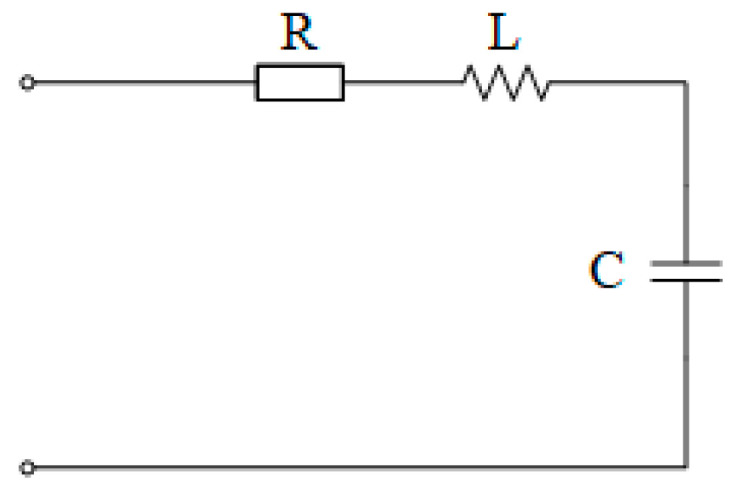
Single-phase equivalent diagram of the primary side in the signal injection method (ignoring the excitation branch).

Due to the symmetry of the three phases, the equivalent circuit of each phase can be expressed as follows (see Figure 2 and Figure 3):

Among them, *R* is the VT leakage resistance, *L* is the VT leakage inductance, and *C* is the single-phase capacitance to ground of the power grid. The total voltage drops generated by the current of each phase across the leakage resistance *R*, leakage reactance *X_L_*, and capacitance reactance *X_C_* to ground of the power grid are *U_a_*, *U_b_*, and *U_c_*, respectively, which satisfy

(4) $${\dot{U}}_{a}={\dot{U}}_{b}={\dot{U}}_{c}$$

(5) $${\dot{U}}_{a}={\dot{I}}_{a}\times \left[R+j(\omega L-\frac{1}{\omega C})\right]$$

Thus, the relationship between the voltage at the open delta terminal of the secondary side and the injected current signal *I*_0_ can be obtained:

(6) $${\dot{U}}_{0}=\frac{{\dot{U}}_{a}+{\dot{U}}_{b}+{\dot{U}}_{c}}{k}=\frac{3{\dot{I}}_{0}\left[R+j(\omega L-\frac{1}{\omega C})\right]}{k^{2}}$$

The dual-frequency method (phasor method) involves injecting two current signals of different frequencies into the secondary side, measuring the magnitude and phase of the returned voltage and current at the open delta terminal of the VT secondary side, and establishing two equations to solve for the unknowns

(7) k23ImU˙01I˙0=ImZ1=ω1L−1ω1Ck23ImU˙02I˙0=ImZ2=ω2L−1ω2C

where Im represents the imaginary part. Z_1_ and Z_2_ stand for the measured equivalent total impedance of the primary side. The solution can be obtained as

(8) C=ω12−ω22ω1ω22ImZ1−ω2ω12ImZ2

In actual measurement, the angular frequencies of the injected signals are known, which are, respectively

(9) $$\left\{\begin{array}{l} \omega_{1}=2\pi f_{1}\\ \omega_{2}=2\pi f_{2} \end{array}\right.$$

The voltage value $U_{0i}$ at the open delta terminal of the VT secondary side, and the phase difference $\theta_{0i}$ between the voltage and current can be measured. Thus

(10) ImZ1=k2U013I0⋅sinθ1ImZ2=k2U023I0⋅sinθ2

Substituting the above equations into the formula, we obtain

(11) $$C=\frac{{f_{1}}^{2}-{f_{2}}^{2}}{2\pi f_{1}f_{2}\cdot \frac{k^{2}}{3I_{0}}\left(f_{2}U_{01}\sin\theta_{1}-f_{1}U_{02}\sin\theta_{2}\right)}$$

### 2.2. Error Analysis

With reference to the error analysis in references [11] and [14], taking the logarithm of both sides of the equation, we obtain

(12) lnC=ln(ω12−ω22)−ln(ω1ω22ImZ1−ω2ω12ImZ2)

‘ln’ stands for natural logarithm. Taking the derivative of the above equation, we obtain

(13) dCC=−ω2dImZ1−ω1dImZ2ω2ImZ1−ω1ImZ2

Thus

(14) ω2ImZ1−ω1ImZ2=ω12−ω22ω1ω2C

Substituting into Equation (13), we have

(15) dC=−ω1ω22dImZ1−ω12ω2dImZ2ω12−ω22⋅C2

The error of the dual-frequency method can be expressed as follows:

(16) dC=E1dImZ1+E2dImZ2

(17) $$E_{1}=\frac{{\omega_{1}}^{2}\omega_{2}C^{2}}{{\omega_{1}}^{2}-{\omega_{2}}^{2}},E_{2}=\frac{{\omega_{2}}^{2}\omega_{1}C^{2}}{{\omega_{1}}^{2}-{\omega_{2}}^{2}}$$

where *dImZ*_1_ and *dImZ*_2_ are the errors of the respective variables *Z*_1_ and *Z*_2_, while *E*_1_ and *E*_2_ are the amplification coefficients of the errors of the respective variables. For different capacitance values, different combinations of ω_1_ and ω_2_ are selected, and the results of the error amplification coefficients are as follows (Table 3):

Analysis of the above table leads to the following conclusions:

(1) When the dual-frequency method is used to measure the system’s capacitance to ground, the error amplification coefficient increases with the increase in the measured capacitance. Therefore, the dual-frequency method has higher accuracy when measuring small capacitance systems.
(2) Within a certain frequency range, the larger the difference between the injected signal frequencies Ω*_1_* and Ω*_2_* selected by the dual-frequency method, the smaller the error amplification coefficient. Thus, the larger the frequency difference in the injected signals, the smaller the measurement error. Therefore, the principle of “one high frequency + one low frequency” should be adopted for frequency selection [14,15].

## 3. Inapplicability of the Traditional Dual-Frequency Method to Systems with High-Resistance Neutral Grounding

**Figure 4:**
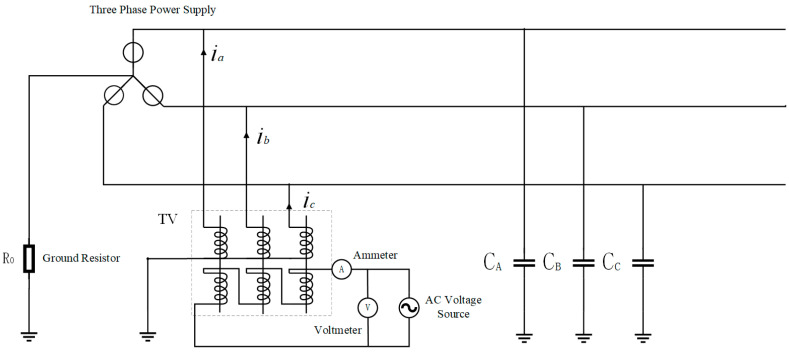
Schematic diagram of the signal injection method in high-resistance grounding systems.

**Figure 5:**
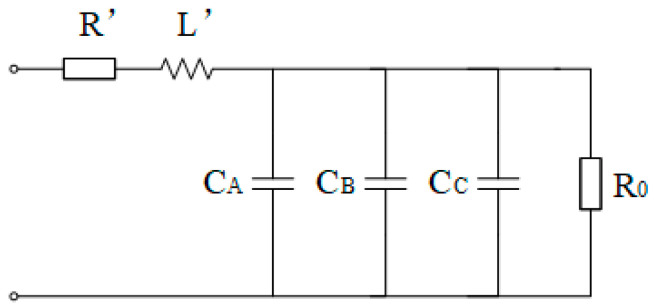
Primary-side equivalent circuit of the high-resistance neutral grounding system.

**Figure 6:**
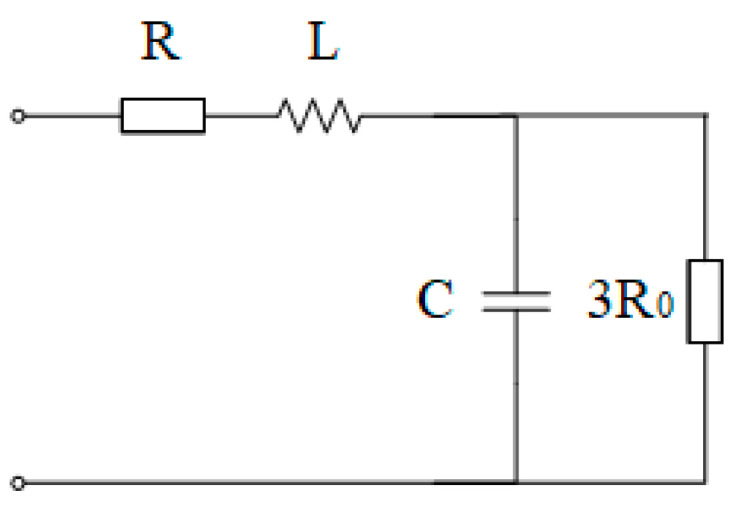
Single-phase equivalent diagram of the primary side in the high-resistance neutral grounding system.

Figure 4 shows a power grid with neutral grounded via high resistance. The impedance is calculated by injecting a signal different from the power frequency into the open delta terminal of the VT secondary side to solve for the capacitance. This signal will induce a corresponding current on the VT primary side (Figure 5). The equivalent circuit of the primary side for the injected non-power frequency signal is as follows (ignoring the excitation branch):

*R’* is the parallel value of the three-phase leakage resistances *R*, *L’* is the parallel value of the three-phase leakage inductances *L*, and *R_0_* is the grounding resistance. Treating *R*_0_ as the parallel combination of three resistors, the value of each resistor is 3*R*_0_, and the single-phase equivalent circuit of the primary side can be drawn as follows (Figure 6):

*C* is the single-phase capacitance to ground of the power grid. It is still defined that the total voltage drops generated by the current of each phase across the leakage resistance *R*, leakage inductance *L*, and the parallel branch of the capacitance to ground *C*, and grounding resistance 3*R*_0_ of the power grid are *U_a_*, *U_b_*, and *U_c_*, respectively.

(18) $${\dot{U}}_{a}={\dot{I}}_{a}\times \left(R+j\omega L+\frac{\frac{1}{j\omega C}\cdot 3R_{0}}{\frac{1}{j\omega C}+3R_{0}}\right)$$

To simplify the formula, we obtain

(19) $${\dot{U}}_{a}={\dot{I}}_{a}\times \left(R+j\omega L+\frac{3R_{0}-j9\omega C{R_{0}}^{2}}{1+9\omega^{2}C^{2}{R_{0}}^{2}}\right)$$

The relationship between the voltage and current at the open delta terminal of the secondary side can be obtained as follows

(20) $${\dot{U}}_{0}=\frac{{\dot{U}}_{a}+{\dot{U}}_{b}+{\dot{U}}_{c}}{k}=\frac{3{\dot{U}}_{a}}{k}=\frac{3{\dot{I}}_{0}}{k^{2}}\cdot \left(R+j\omega L+\frac{3R_{0}-j9\omega C{R_{0}}^{2}}{1+9\omega^{2}C^{2}{R_{0}}^{2}}\right)$$

Which is as follows

(21) $$\frac{k^{2}}{3}\cdot \frac{{\dot{U}}_{0}}{{\dot{I}}_{0}}=R+j\omega L+\frac{3R_{0}-j9\omega C{R_{0}}^{2}}{1+9\omega^{2}C^{2}{R_{0}}^{2}}$$

To simplify the calculation, only the imaginary part is considered

(22) k23ImU˙0I˙0=ImZ=ωL−9ωCR021+9ω2C2R02

Z stands for the measured equivalent total impedance of the primary side. Using the idea of the dual-frequency method, it can be obtained from Formula (22)

(23) ImZ1=ω1L−9ω1CR021+9ω12C2R02ImZ2=ω2L−9ω2CR021+9ω22C2R02

Eliminating the parameter *L*, we obtain

(24) $$Z_{1}\omega_{2}{-Z}_{2}\omega_{1}=9\omega_{1}\omega_{2}R_{0}^{2}C\cdot \left(\frac{1}{1+9{\omega_{2}}^{2}R_{0}^{2}C^{2}}-\frac{1}{1+9{\omega_{1}}^{2}R_{0}^{2}C^{2}}\right)$$

Finally

(25) $$Z_{1}\omega_{2}{-Z}_{2}\omega_{1}=\frac{81\left({\omega_{1}}^{2}-{\omega_{2}}^{2}\right)\omega_{1}\omega_{2}R_{0}^{4}C^{2}}{\left(1+9{\omega_{1}}^{2}R_{0}^{2}C^{2}\right)\left(1+9{\omega_{2}}^{2}R_{0}^{2}C^{2}\right)}$$

This is a quartic equation in one variable, which is difficult to solve and may have multiple solutions or no real solutions, bringing great difficulties to the solution of the capacitance *C*. Therefore, the traditional dual-frequency method is not applicable to the online measurement of the capacitance to ground of systems with neutral grounded via high resistance.

## 4. Proposal of the High–Low Frequency Combination Method

This paper proposes a signal injection method based on the high–low frequency combination to realize the online measurement of the capacitance to ground of medium-voltage power systems with neutral grounded via high resistance. For the injection position of the signal, three-phase transformers can be used to inject signals from the three-phase power grid, or a single-phase transformer can be used to inject signals from the neutral point. In the following, this paper derives the calculation formula for signal injection from the neutral point.

**Figure 7:**
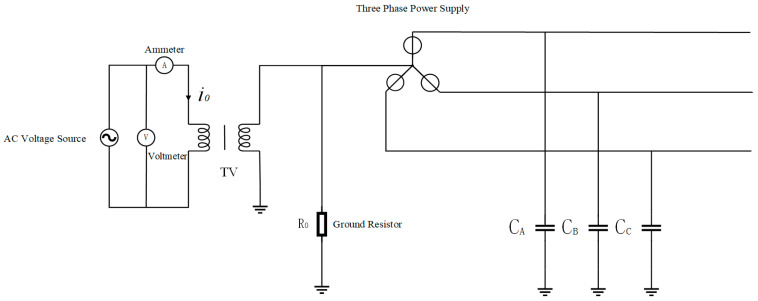
Schematic diagram of capacitance measurement in power grids by the neutral point signal injection method.

**Figure 8:**
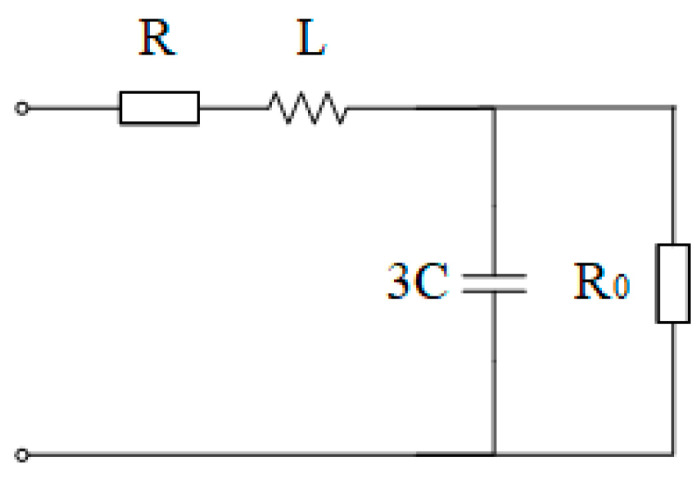
Primary-side equivalent diagram of the high-resistance neutral grounding system.

Figure 7 shows a power grid with neutral grounded via high resistance. A voltage signal is injected into the neutral point of the power grid through a single-phase voltage transformer, and a corresponding induced current will be induced on the VT primary side. The equivalent circuit of the primary side for the injected signal is the same as that in Figure 8. If the three-phase capacitances $C_{A}=C_{B}=C_{C}=C$, the equivalent circuit is as follows:

*R* is the transformer leakage resistance, *L* is the transformer leakage inductance, and *R*_0_ is the grounding resistance value. The voltage drop on the high-voltage side of the transformer is defined as *U*_1_, and the current in the circuit is *I*_1_. Then

(26) $${\dot{U}}_{1}={\dot{I}}_{1}\times \left[R+j\omega L+\frac{\frac{1}{3j\omega C}\cdot R_{0}}{\frac{1}{3j\omega C}+R_{0}}\right]$$

To simplify it, we obtain

(27) $${\dot{U}}_{1}={\dot{I}}_{1}\times \left[R+j\omega L+\frac{R_{0}-3j\omega C{R_{0}}^{2}}{1+9\omega^{2}C^{2}{R_{0}}^{2}}\right]$$

Converting the voltage and current to the low-voltage side, we have the following

(28) $$\frac{k^{2}{\dot{U}}_{0}}{{\dot{I}}_{0}}=R+j\omega L+\frac{R_{0}-3j\omega C{R_{0}}^{2}}{1+9\omega^{2}C^{2}{R_{0}}^{2}}$$

When the frequency of the injected signal is sufficiently high, only the inductive reactance can be considered while ignoring the other three impedances. Thus

(29) $$L=\frac{k^{2}U_{0}}{\omega I_{0}}$$

Taking the imaginary part of the formula, we obtain

(30) Imk2U˙0I˙0=ωL−3ωCR021+9ω2C2R02

Let

(31) A=ωL−Imk2U˙0I˙0

The original formula can be simplified to

(32) $$9A\omega^{2}{R_{0}}^{2}C^{2}-3\omega {R_{0}}^{2}C+A=0$$

Thus, the solution is

(33) $$C=\frac{R_{0}\pm \sqrt{{R_{0}}^{2}-4A^{2}}}{6A\omega R_{0}}$$

Take Formula (31) into consideration, we have

(34) $$C=\frac{R_{0}\pm \sqrt{{R_{0}}^{2}-4(\omega L-\frac{k^{2}U_{0}}{I_{0}}\cdot \sin\theta {)}^{2}}}{6\omega R_{0}(\omega L-\frac{k^{2}U_{0}}{I_{0}}\cdot \sin\theta )}$$

To measure the capacitance to ground of the power grid using the high–low frequency combination method with signal injection from the neutral point, firstly, we need to inject a sufficiently high-frequency signal into the secondary side, measure the current amplitude on the transformer secondary side, and solve for the transformer leakage inductance *L* using the above formula. Then, a low-frequency signal needs to be injected into the secondary side, the amplitude of the current feedback and the phase difference between the voltage and current at the open delta terminal of the secondary side are measured, and the formula is used to solve for C.

## 5. Frequency Selection and Simulation Verification

### 5.1. Model Establishment

A ship medium-voltage power grid model was established using the MATLAB (2020)/Simulink module, with the neutral point grounded via high resistance. Signals were injected through the three-phase power grid, and a three-phase transformer was used instead of the voltage transformer (VT) to simulate the uncharged state of the power grid. Signals of different frequencies were injected, and the proposed high–low frequency combination method was used to solve for the VT leakage inductance *L* and capacitance to ground *C*. These values were compared with the set values to explore the most suitable frequency selection scheme.

**Figure 9:**
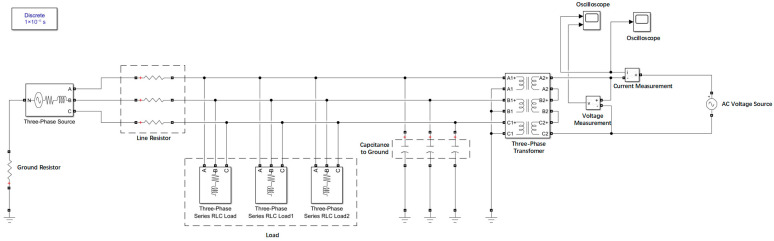
Simulation model of the ship’s medium-voltage power grid.

The following simulation model was established (Figure 9):

The parameters of each module were set as follows:

(1) Power grid voltage (offline measurement): 0 V.
(2) Single-phase capacitance to ground in different experiments: 1 μF, 5 μF, 10 μF, 15 μF, 20 μF.
(3) Voltage source of the measurement circuit: 1 V (variable frequency).
(4) Cable resistance: 5 Ω.
(5) Grounding resistance: 1 kΩ.

### 5.2. Selection of High-Frequency Signals

For the selection of the high-frequency signal frequency, this paper combines the basic principle of frequency selection in the traditional dual-frequency signal injection method and the conclusions from the error analysis in Section 2. The frequency of the high-frequency signal should be so large that the system inductive reactance *X_L_* is much larger than the VT leakage resistance *R*, additional impedance *R*_1_, and variable capacitance reactance *BX_C_*, thereby greatly reducing the calculation workload within a reasonable error range. Under power frequency conditions, the leakage inductive reactance is approximately several thousand ohms, the capacitance reactance *BX_C_* is approximately several hundred ohms, the equivalent resistance *R*_1_ caused by the equivalent impedance grounding resistance is approximately several tens of ohms, while the VT leakage resistance *R* is approximately tens of thousands of ohms [16], which does not meet the condition of only considering the leakage inductive reactance while ignoring *R*, *R*_1_, and *BX_C_*. If the frequency is increased to several thousand hertz, the difference between the inductive reactance and the capacitive reactance is 4–5 orders of magnitude, the inductive reactance is a hundred times larger than the leakage resistance, and 8 orders of magnitude larger than the additional resistance caused by the grounding resistance. In this case, the capacitive reactance and resistance can be completely ignored. In this paper, the simulation model established in the previous section was used to inject high-frequency signals of different frequencies to verify the correctness of this method and find a suitable frequency.

**Figure 10:**
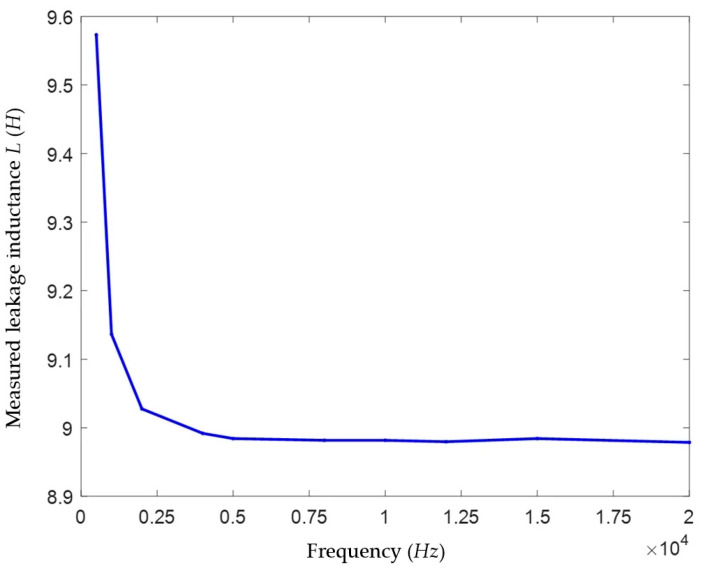
High-frequency signal test results.

In this paper, the single-phase capacitance to ground in the model above was set to 10 μF, and voltage signals of different frequencies (500–20,000 Hz) with a fixed amplitude of 1 V were injected. The loop current *I*_0_ was measured using an ammeter and an oscilloscope module, and the leakage inductance *L* was solved. The results are shown in Table 4 and Figure 10.

According to the experimental measurement results in Reference [11], the leakage inductance of the measured JSZW-10 type voltage transformer is approximately 9 H. It can be seen from Figure 10 and Table 4 that among the transformer leakage inductance values measured using high-frequency signals of 500–20,000 Hz, the measured values are around 9 H when the signal frequency is above 2000 Hz and basically stabilize at approximately 8.98 H after 5000 Hz. According to the analysis before, theoretically, the larger the high frequency, the smaller the error of the solved *L* value. Therefore, it can be considered that the VT leakage inductance values measured at high frequencies of 5000 Hz and above are relatively accurate, and the measured value is taken as 8.98 H.

On the other hand, since the voltage transformer also has a small capacitance to ground, to avoid the injected voltage signal being filtered out by the VT’s capacitance to ground, this paper should also minimize the frequency of the injected signal under the premise of ensuring a relatively accurate measurement of the VT leakage inductance. Considering the above factors, the high-frequency signal frequency was selected as 5000 Hz in this paper.

### 5.3. Selection of Low-Frequency Signals

Since the injection of low-frequency signals directly solves for the system’s capacitance to ground, the correct selection of the low-frequency signals has a crucial impact on the accuracy of the measurement results. For systems with large capacitance to ground, if the selected low frequency is too high, the corresponding capacitance reactance *BX_C_* is much smaller than the VT leakage reactance *X_L_*, and the measurement error of the phase difference *θ* will lead to a large deviation in the calculated value of *BX_C_*, resulting in excessive calculation errors, which means, for large capacitance systems, the low frequency should be selected to be relatively small. However, signals with excessively low frequencies will make the excitation branch current unignorable, so the low frequency cannot be selected to be too low.

**Figure 11:**
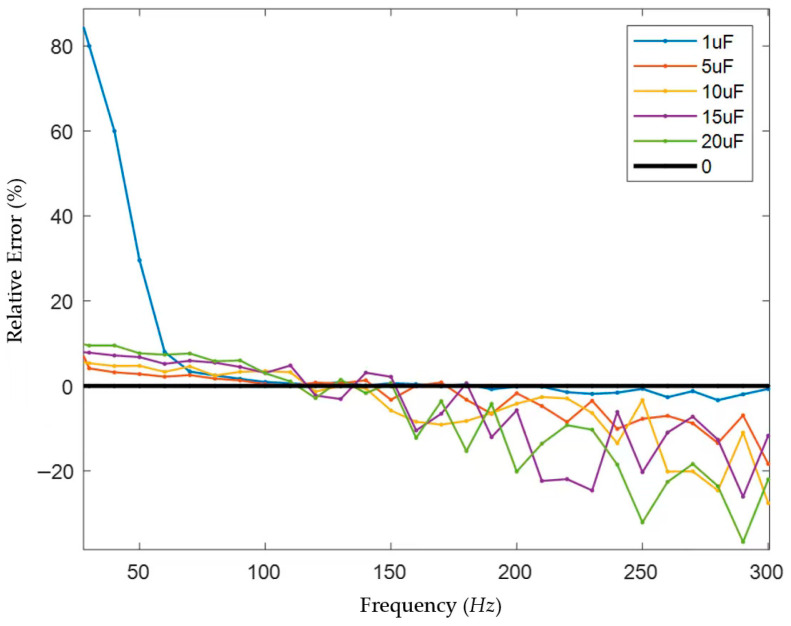
Error in capacitance measurement results.

In this paper, the single-phase capacitance to ground in the model above was set to 1 μF, 5 μF, 10 μF, 15 μF, and 20 μF, respectively. Voltage signals of different frequencies with a fixed amplitude of 1 V were injected. The loop current *I*_0_ and the phase difference *θ* between the voltage and current were measured using an ammeter, a voltmeter, and an oscilloscope module, and the capacitance to ground C was solved. The results are shown in Figure 11.

In general, the capacitance measurement error is small in the range of 70 Hz to 150 Hz. When the single-phase capacitance to ground is 1 μF, the measurement error is stable within ±5% in the frequency range of 70–330 Hz, with the smallest error near 110 Hz; when the single-phase capacitance to ground is 5 μF, the measurement error is stable within ±5% in the frequency range of 30–180 Hz, with the smallest error near 110 Hz; when the single-phase capacitance to ground is 10 μF, the measurement error is stable within ±5% in the frequency range of 40–140 Hz, with the smallest error near 130 Hz; when the single-phase capacitance to ground is 15 μF, the measurement error is stable within ±5% in the frequency range of 90–150 Hz, with the smallest error near 120 Hz; when the single-phase capacitance to ground is 20 μF, the measurement error is stable within ±5% in the frequency range of 100–150 Hz, with the smallest error near 110 Hz (although the error at 150 Hz is smaller, it is unstable, and the errors at adjacent frequencies are large). Since the capacitance of the medium-voltage power systems in large-scale ships is generally above 4 μF and below 10 μF, and it is necessary to avoid interference from the 50 Hz power frequency, the low frequency was selected as 120 Hz after careful consideration.

Since the injection of low-frequency signals is directly related to solving the value of the system’s capacitance to ground, whether the low-frequency signal is correctly selected or not plays a crucial role in the accuracy of the measurement results. For systems with a relatively small capacitance to ground, even if the low-frequency selection frequency is relatively high, the corresponding capacitive reactance BX_C_ is also relatively large, and its size is comparable to that of the VT leakage reactance X_L_. The slight measurement error of the phase angle difference will not cause a large deviation in the calculated value of the capacitive reactance BX_C_. For systems with a large capacitance to ground, if the low-frequency selection is too high, the corresponding capacitive reactance value BX_C_ is much smaller than the VT leakage reactance value X_L_. The measurement error of the phase angle difference will cause a large deviation in the calculated value of BX_C_, thereby resulting in excessive calculation error.

The above research also verified through simulation that the proposed high–low frequency combination method can accurately measure the capacitance to ground of the medium-voltage power systems with neutral grounded via high resistance offline under the appropriate frequency selection.

### 5.4. Simulation Verification of Online Measurement

In the previous section, while determining the frequency selection strategy through simulation, this paper also verified the accuracy of the high–low frequency combination method for offline measurement of the system’s capacitance to ground under the appropriate frequency selection scheme. In this section, the feasibility of the method for online measurement is verified through live measurement, providing a basis for the online real-time measurement of the capacitance to ground of ship medium-voltage power systems, thereby realizing the real-time adjustment of grounding resistance parameters.

**Figure 12:**
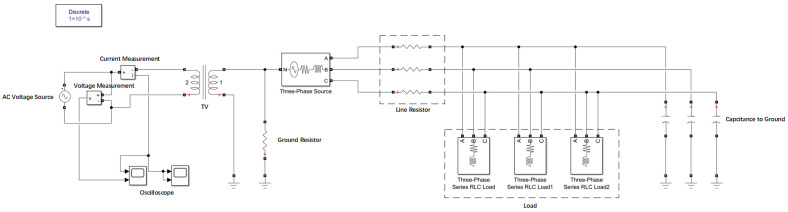
Simulation model of the ship’s medium-voltage power grid.

The following is the simulation verification of online measurement using the neutral point signal injection method, and the following power grid model was established (Figure 12):

In this model, a single-phase transformer is used to inject signals at the neutral point of the power grid, and the returned current value is measured for calculation.

When the power grid was live, the single-phase capacitance to ground was set to 1 μF, 5 μF, 10 μF, 15 μF, and 20 μF, respectively, and the frequency of 5000/120 Hz was selected for measurement. The results are as follows (Table 5):

From Table 5, it can be observed that in the range of single-phase capacitance to ground of 1–20 μF, the high–low frequency combination method with signal injection at the neutral point and frequency selection of 5000/120 Hz is used for capacitance measurement. The relative error between the measurement results and the set values is within 5%. Thus, it can be verified that when the power grid is live, the high–low frequency combination method with signal injection at the neutral point can realize the measurement of the capacitance to ground of the medium-voltage systems with neutral grounded via high resistance.

## 6. Experimental Verification

To further verify the correctness and feasibility of the high–low frequency combination method, this chapter adopts a scaled-down experiment for experimental verification of capacitance measurement.

### 6.1. Scaled-Down Platform and Experimental Scheme

Due to the limitations of experimental conditions, a 380 V power grid scaled-down experiment was adopted in this paper. The instruments used are as follows:

(1) SG-2/0.5-380/380-50 three-phase isolation transformer (Wuhan Newrock Electric Technology Co. Ltd., Wuhan, China).
(2) JSZXT8-6.3TH voltage transformer (rated voltage ratio 3600/60/60, leakage impedance parameters obtained through short-circuit test: *Z* = 6970.8 Ω, *R* = 6555.0 Ω, *L* = 18.9 H) (Ningbo Tonghe Transformer Co., Ltd., Ningbo, China).
(3) Tektronix AFG 3021B signal generator.
(4) Current probe.
(5) TBS2000B digital oscilloscope.
(6) UT56 digital multimeter.
(7) CBB capacitors (0.33 μF, 2.2 μF, 4.7 μF).

**Figure 13:**
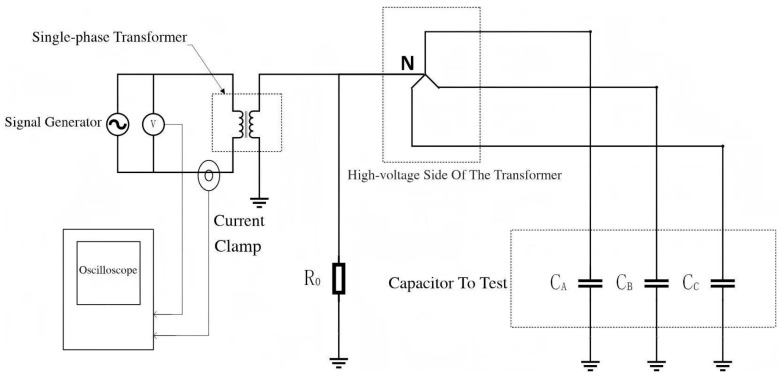
Experimental wiring diagram.

**Figure 14:**
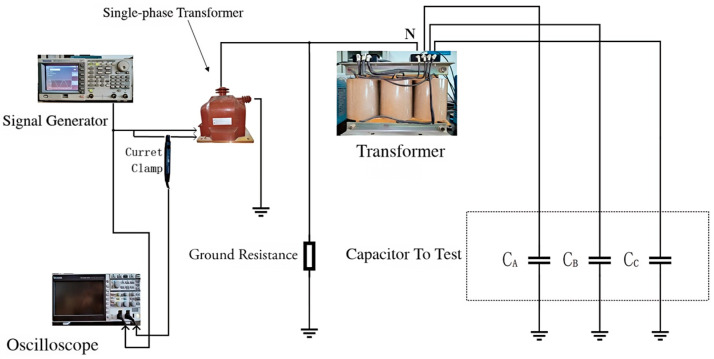
Actual experimental wiring diagram.

The platform was established as shown in the following Figure 13 and Figure 14:

In this experiment, capacitors were used instead of the distributed capacitance of cables. Considering that the estimated capacitance to ground of an actual medium-voltage ship is approximately 5 μF [17], the single-phase capacitance to ground was set to 0.33 μF, 2.2 μF, and 4.7 μF, respectively. The grounding resistance was configured according to the principle that the resistance value is equal to or slightly less than 1/3 of the single-phase capacitance reactance to ground, and grounding resistors of 247 Ω, 495 Ω, and 3310 Ω were connected, respectively. A signal generator was used to inject high-frequency signals with frequencies of 1 k–10 kHz and a voltage amplitude of 5 V into the secondary winding of the transformer, and the leakage inductance value *L* of the transformer was calculated using the formula. Then, voltage signals with frequencies of 20 Hz, 100 Hz, 120 Hz, and 150 Hz were injected, and the formula was used to solve for the capacitance to ground, *C*.

### 6.2. Experimental Data

#### 6.2.1. Injecting High-Frequency Signals to Measure the Leakage Inductance L

The experiment was carried out according to the experimental scheme in Section 5.1. The feedback current was measured when high-frequency signals were input, and the leakage inductance value *L* of the transformer was calculated using the formula as follows (Table 6):

When the injected signal frequency is above 3000 Hz, the measured *L* value is basically stable at approximately 19.1 H, which is basically consistent with the result *L* = 18.9 H obtained from the short-circuit test, with an error of 1.05%. When the high frequency is selected as 5000 Hz, the measured value is 19.1 H, which can verify the correctness of the method for calculating the transformer leakage inductance using the high frequency of 5000 Hz and the rationality of frequency selection. The value of 19.1 H was used as the calculated value in the subsequent calculations.

#### 6.2.2. Injecting Low-Frequency Signals to Measure C

When low-frequency signals were input, the feedback current and the phase difference between voltage and current were measured at different frequencies, and *C* was calculated using the formula.

1. Connecting a 0.33 μF capacitor per phase

A multimeter was used to measure the parameters of the three capacitors, and the measured capacitances were 0.33 μF, 0.32 μF, and 0.33 μF, respectively. The average value was calculated as 0.33 μF.

The results measured by injecting low-frequency signals are as follows (Table 7):

2. Connecting a 2.2 μF capacitor per phase

The results measured by injecting low-frequency signals are as follows (Table 8):

3. Connecting a 4.7 μF capacitor per phase

The results measured by injecting low-frequency signals are as follows (Table 9):

### 6.3. Analysis of Measurement Results

**Figure 15:**
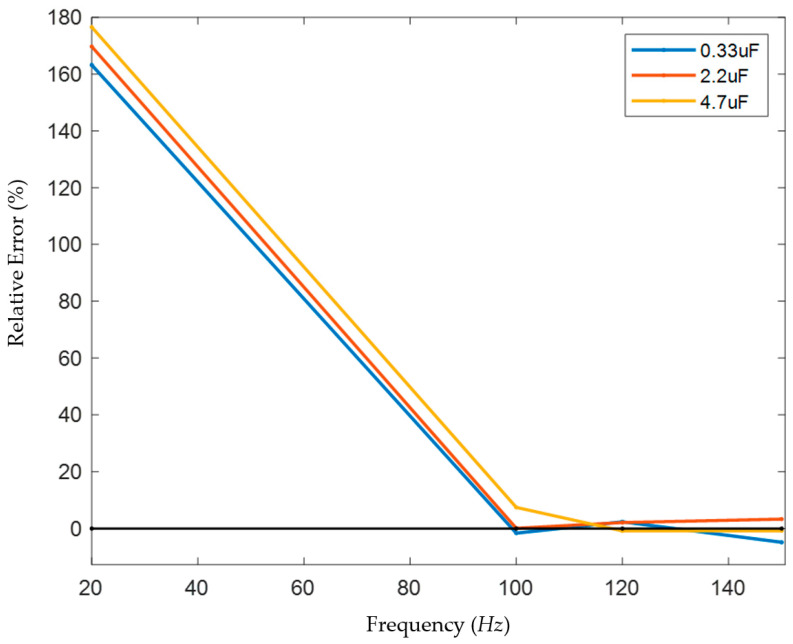
Analysis of measurement results.

The measurement errors of the measurement results are plotted as follows (Figure 15):

It can be found from the measurement results that the high–low frequency combination method can accurately measure the large system’s capacitance to ground under the appropriate frequency scheme. When the low frequency is selected in the range of 100–150 Hz, the error can be kept within 10%. When the low frequency is selected as 20 Hz, the measured capacitance values are all larger than the actual values.

According to DNV’s Rules for Classification: Ships, “The neutral point of the system is grounded through a resistor, and the resistance value is equal to or slightly less than one-third of the reactance value between one phase and the ground.” Therefore, an error of less than 5% is allowed, which does not affect the final parameter setting, and this method has a higher measurement accuracy compared to the method proposed in the references. It can be verified that under the frequency scheme of 5000/120 Hz, the high–low frequency combination method can accurately measure the capacitance to ground of systems with neutral grounded via high resistance and large capacitance to ground.

## 7. Conclusions

To address the critical practical demand for accurate and real-time online measurement of the capacitance to ground in medium-voltage (MV) power grids of large-scale ships, this study systematically investigates existing capacitance measurement methods and proposes an optimized solution. Through simulation verification and experimental verification, it is finally proved that the proposed method can accurately realize the online measurement of the capacitance to ground of the medium-voltage power system with neutral grounded via high resistance.

### 7.1. Key Research Findings

(1) Methodological Innovation: Traditional signal injection methods are unsuitable for MV systems with high-resistance neutral grounding—either due to narrow applicability (small capacitance only) or complex solving processes (quartic equations with multiple solutions). The proposed high–low frequency combination method overcomes these limitations by integrating the dual-frequency method and high-frequency method, enabling direct and reliable calculation of capacitance without complex equation solving.
(2) Optimal Frequency Scheme: Through simulation and experimental optimization, the frequency combination of 5000 Hz (high frequency) + 120 Hz (low frequency) is determined as optimal:

The 5000 Hz high-frequency signal effectively isolates the transformer leakage inductance (*L*) by ignoring other impedances, ensuring accurate extraction of L for error correction.

The 120 Hz low-frequency signal avoids interference from 50 Hz power frequency and minimizes phase measurement errors, achieving stable results for capacitances ranging from 1 μF to 20 μF.

(3) Measurement Accuracy Verification: Both simulation and scaled-down experiments confirm that the proposed method delivers high precision:

Online measurement relative errors are within ±5% for capacitances of 1–20 μF (covering the typical capacitance range of large ship MV systems: 4–10 μF).

Experimental results with actual voltage transformers (leakage inductance ≈ 19.1 H) show errors < 3% for target capacitances (0.33 μF, 2.2 μF, 4.7 μF), verifying robustness against parasitic parameters and hardware constraints.

### 7.2. Practical Applications

By providing accurate online capacitance data, the method enables dynamic adjustment of high-resistance grounding parameters, ensuring the grounding arc is reliably extinguished. The method uses conventional signal injection hardware (signal generators, oscilloscopes, current probes) and avoids complex algorithms, enabling easy integration into existing ship monitoring systems with low retrofitting costs.

## Figures and Tables

**Table 1:** Comparative analysis of signal injection methods.

Measurement Method	Measurement Principle	Advantages	Disadvantages
Three-frequency Method	Measuring zero-sequence impedance	Good safety, convenient measurement	Only applicable to systems with small capacitance to ground; difficult to select frequency combinations
Dual-frequency Method	Measuring zero-sequence impedance	Wider application range compared with the three-frequency method	Difficult to select frequency combinations
Resonance Method	Measuring zero-sequence impedance and the resonance principle	Good safety, convenient measurement	Only applicable to systems with small capacitance to ground; difficult to select frequency combinations

**Table 2:** Influence of the voltage transformer on measurement.

Comparison Indicator	Three-Frequency Method	Dual-Frequency Method	Resonance Method
Single-phase Capacitance Measurement Range	<2 μF	<4 μF	<10 μF
Influence of VT Primary Side Leakage Resistance	Significant	None	None
Influence of VT Primary Side Leakage Inductance	Significant	Minor	Significant
Influence of VT Secondary Side Leakage Resistance	Significant	None	None
Influence of VT Secondary Side Leakage Inductance	Significant	Minor	Minor

**Table 3:** Error amplification coefficients of the dual-frequency method.

C(μF)	1	1	1	10	10	10	20	20	20
$\omega_{1}∕\omega_{2}(\frac{rad}{s})$	10/20	10/100	10/5000	10/20	10/100	10/5000	10/20	10/100	10/5000
$E_{1}$	−6.67 × 10^−12^	−1.01 × 10^−12^	−0.02 × 10^−12^	−6.67 × 10^−10^	−1.01 × 10^−10^	−0.02 × 10^−10^	−26.68 × 10^−10^	−4.04 × 10^−10^	−0.08 × 10^−10^
$E_{2}$	−13.33 × 10^−12^	−10.10 × 10^−12^	−10.00 × 10^−12^	−13.33 × 10^−10^	−10.10 × 10^−10^	−10.00 × 10^−10^	−53.32 × 10^−10^	−40.40 × 10^−10^	−40.00 × 10^−10^

**Table 4:** High-frequency signal test results.

Frequency (Hz)	*I*_1_ (A)	*L* (H)
500	0.33250	9.573238
1000	0.17420	9.136342
2000	0.08815	9.027514
4000	0.04425	8.991812
5000	0.03543	8.984199
8000	0.02215	8.981664
10,000	0.01772	8.981664
12,000	0.01477	8.979637
15,000	0.01181	8.984199
20,000	0.00886	8.978623

**Table 5:** Online measurement results of different capacitance values.

Capacitance Value *C* (μF)	*I*_1_ (mA)	*L* (H)	*I*_2_ (A)	*θ* (°)	Measured Capacitance Value *C* (μF)	Relative Error
1.00	5.993	19.1209	0.2566	88.819	0.991	0.90%
5.00	5.993	19.1209	0.2515	89.424	5.051	1.02%
10.00	5.993	19.1209	0.2507	89.467	10.214	2.14%
15.00	5.993	19.1209	0.2505	89.467	15.505	3.36%
20.00	5.993	19.1209	0.2503	89.467	20.898	4.49%

**Table 6:** Online measurement results of leakage inductance by the neutral point injection method.

Frequency (Hz)	*I*_1_ (A)	*L* (H)
1000	0.1600	17.90495
2000	0.0765	18.72413
3000	0.0502	19.02252
4000	0.0376	19.04782
5000	0.0301	19.09861
6000	0.0250	19.09861
7000	0.0214	19.12411
8000	0.0187	19.14968
9000	0.0167	19.06049
10,000	0.0150	19.09861

**Table 7:** Online measurement results when connecting a 0.33 μf capacitor.

Grounding Resistance R_0_ (Ω)	Capacitance Value *C* (μF)	Signal Voltage (V)	Signal Frequency (Hz)	Feedback Current Value *I*_2_ (A)	Phase Difference *θ* (°)	Measured Capacitance Value *C* (μF)	Relative Error
3310	0.33	0.31	20	0.1270	4.90	0.8687	163.23%
		0.486	100	0.1290	52.01	0.3247	−1.61%
		0.599	120	0.1313	53.92	0.3378	2.37%
		0.678	150	0.1312	65.98	0.3141	−4.831%

**Table 8:** Online measurement results when connecting a 2.2 μf capacitor.

Grounding Resistance R_0_ (Ω)	Capacitance Value *C* (μF)	Signal Voltage (V)	Signal Frequency (Hz)	Feedback Current Value *I*_2_ (A)	Phase Difference *θ* (°)	Measured Capacitance Value *C* (μF)	Relative Error
495	2.23	0.296	20	0.1085	12.67	6.0145	169.71%
		0.511	100	0.1300	56.56	2.2322	0.10%
		0.592	120	0.1315	61.43	2.2770	2.11%
		0.699	150	0.1314	68.86	2.3040	3.32%

**Table 9:** Online measurement results when connecting a 4.7 μf capacitor.

Grounding Resistance R_0_ (Ω)	Capacitance Value *C* (μF)	Signal Voltage (V)	Signal Frequency (Hz)	Feedback Current Value *I*_2_ (A)	Phase Difference *θ* (°)	Measured Capacitance Value *C* (μF)	Relative Error
247	4.61	0.283	20	0.1242	16.128	12.7501	176.57%
		0.500	100	0.1286	58.32	4.9536	7.45%
		0.575	120	0.1289	63.072	4.5718	−0.83%
		0.700	150	0.1288	66.42	4.5750	−0.76%

## Data Availability

The original contributions presented in this study are included in the article. Further inquiries can be directed to the corresponding author.
